# Lymphocyte Micronucleus Formation Is Driven by Inflammation‐Induced Oxidative DNA Damage in Oesophageal Cancer Development

**DOI:** 10.1002/ijc.70494

**Published:** 2026-04-25

**Authors:** Kathryn Munn, Rachel Lawrence, Hasan Haboubi, Hamsa Naser, Kate Hurlow, Ume‐Kulsoom Shah, Lisa Williams, Sarah Gwynne, Owen Bodger, Jiri Zavadil, Francois Virard, Shareen Doak, Laura E. Thomas, Gareth Jenkins

**Affiliations:** ^1^ In Vitro Toxicology Group Swansea University Medical School, Swansea University Swansea UK; ^2^ Centre for Biomarkers and Biotherapeutics, Barts Cancer Institute, Queen Mary University of London London UK; ^3^ Department of Gastroenterology Llandough Hospital, Cardiff and Vale University Health Board Cardiff UK; ^4^ Department of Gastroenterology Singleton Hospital, Swansea Bay University Health Board Swansea UK; ^5^ Department of Oncology Singleton Hospital, Swansea Bay University Health Board Swansea UK; ^6^ International Agency for Research on Cancer, Epigenomics and Mechanisms Branch Lyon France; ^7^ University Claude Bernard Lyon 1 INSERM U1052–CNRS UMR5286, Centre Léon Bérard Lyon France

**Keywords:** circulating biomarkers, DNA damage, micronucleus, oesophageal adenocarcinoma, oxidative stress

## Abstract

We investigated mechanisms underlying circulating DNA damage across the gastro‐oesophageal reflux disease (GORD), Barrett's oesophagus (BO) and oesophageal adenocarcinoma (OAC) spectrum using the lymphocyte micronucleus (MN) assay. MN frequency (MN%) was quantified in lymphocytes from healthy volunteers (HVs), GORD, BO and OAC patients, alongside plasma biomarkers of inflammation and oxidative stress. Ex vivo challenge assays assessed lymphocyte responses to hydrogen peroxide (H_2_O_2_), vinblastine and the bile acid deoxycholic acid (DCA). Tissue‐based NF‐κB expression was also correlated with MN levels. OAC patients exhibited significantly elevated MN% compared with HVs (*p* < 0.001), GORD (*p* < 0.001) and BO (*p* = 0.016). OAC lymphocytes showed increased sensitivity to vinblastine relative to HVs (*p* = 0.025) and GORD patients (*p* = 0.033). Higher baseline MN% correlated with reduced inducible MN formation following H_2_O_2_ or DCA, suggesting altered oxidative stress responses. MN% also associated with plasma 8‐OHdG, IL‐8 and 2′3′‐cGAMP, and with reduced oesophageal tissue IκB, indicating activation of oxidative and cGAS‐STING/NF‐κB signalling. These findings highlight systemic aneugenic and oxidative stress processes that contribute to lymphocyte MN formation in OAC and suggest that MN% may serve as a minimally invasive indicator of inflammation‐linked genomic instability. Further investigation is needed to determine its relevance to OAC progression.

## Abstract (highlights)

What's new?

Chromosomal instability is a hallmark of cancer, but minimally invasive ways to detect systemic DNA damage and cancer risk remain limited. Here, using patient blood samples, the authors investigated lymphocyte micronuclei (MN) frequency as a potential marker of DNA damage across the progression from gastroesophageal reflux disease to Barrett's esophagus and esophageal adenocarcinoma (OAC). MN levels were significantly elevated specifically in OAC and were linked to oxidative stress, inflammatory signaling, and altered responses to cell damage. The results suggest that MN frequency reflects systemic genomic instability and could be a minimally invasive marker for inflammation‐driven carcinogenesis and OAC progression.

## Graphical abstract

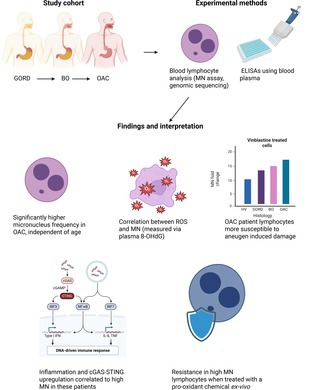

**Abbreviations**
BMI: body mass index
BO: Barrett's oesophagus
CBPI: cytokinesis‐block proliferation index
cGAMP: cyclic GMP‐AMP
cGAS: cyclic GMP‐AMP synthase
CIN: chromosomal instability
CytoB: cytochalasin B
DCA: deoxycholic acid
ELISA: enzyme‐linked immunosorbent assay
FBS: foetal bovine serum
GORD: gastro‐oesophageal reflux disease
HV: healthy volunteers
MN: micronuclei
MN%: micronucleus frequency
OAC: oesophageal adenocarcinoma
PBMCs: peripheral blood mononuclear cells
PHA: phytohemagglutinin
RONS: reactive oxygen and nitrogen species
STING: Stimulator of Interferon Genes

## 1 Introduction

Genomic instability drives a growth advantage and clonal dominance in cancer cells, aligning with the well‐recognised hallmarks of cancer [1]. Chromosomal instability (CIN) is prevalent in cancer genomes [1] and profiling DNA damage at the chromosome level may identify individuals with a predisposition to chromosomal damage and carcinogenesis. The lymphocyte cytokinesis‐block micronucleus (CBMN) test detects aneugenic and clastogenic DNA damage in somatic cells in a minimally invasive manner. Micronuclei (MN) in peripheral blood lymphocytes reflect systemic genotoxic exposure or an underlying susceptibility to chromosomal damage [2]. Although MN have traditionally been used for biomonitoring environmental exposures, emerging evidence suggests they can also result from endogenous processes relevant to cancer development [3]. As previous studies have linked elevated lymphocyte MN levels with a cancer diagnosis, we aimed here to investigate whether such a relationship is also observed in oesophageal adenocarcinoma (OAC) and explore the mechanisms behind MN formation in this context.

**FIGURE 1:**
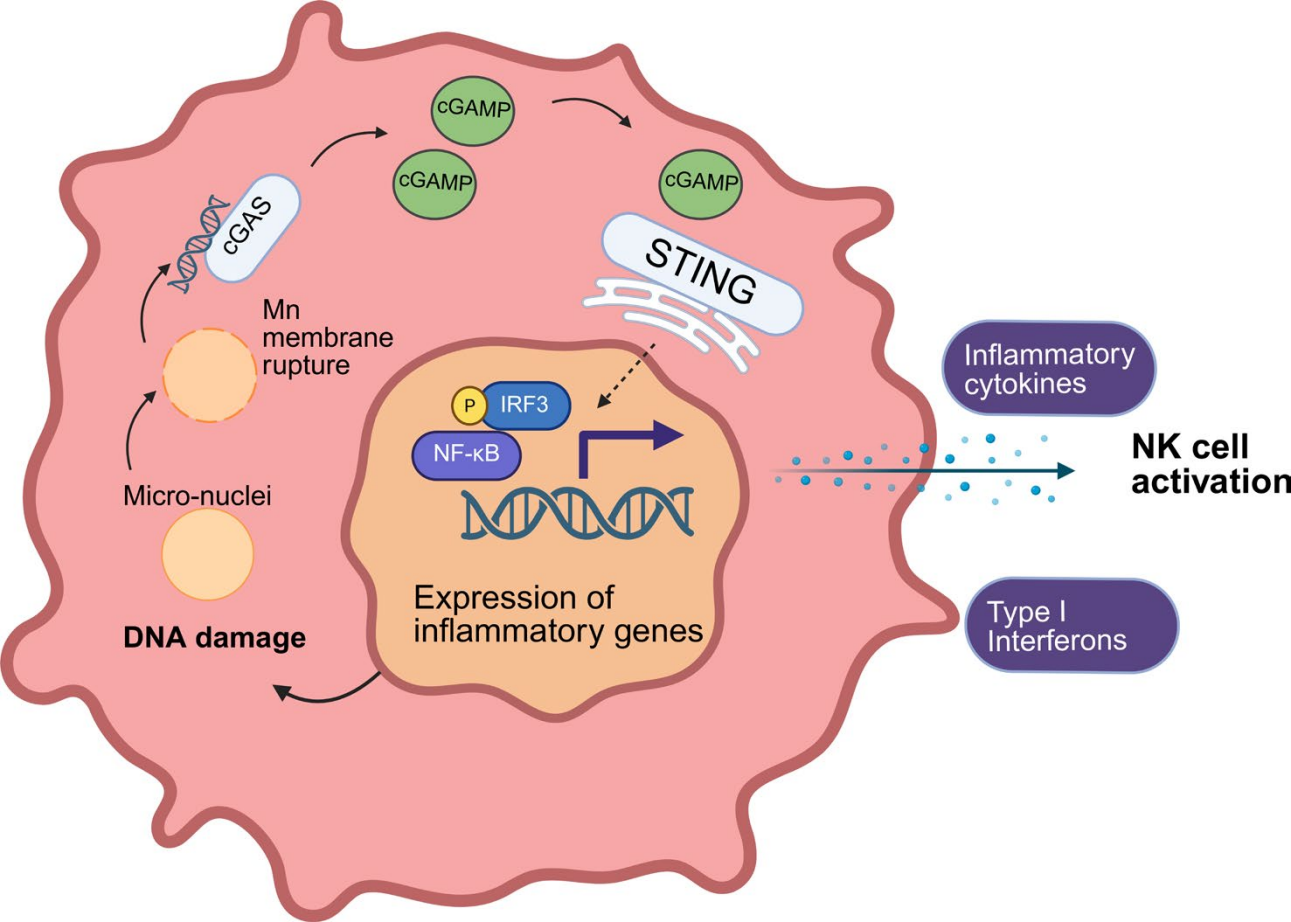
The cGAS‐STING pathway in micronucleated cells. Upon micronuclear (MN) envelope rupture, cyclic GMP‐AMP synthase (cGAS) is activated, catalysing cyclic GMP‐AMP (cGAMP) production. cGAMP binds to stimulator of interferon genes (STING), recruiting kinases IKK and TBK1. TBK1 phosphorylates IRF3, promoting Type 1 interferon gene transcription, while IKK phosphorylates IKB, leading to NF‐kB translocation and subsequent pro‐inflammatory cytokine transcriptional activation. Created using BioRender.com.

Recent evidence has highlighted the capacity of MN to activate the pro‐inflammatory cGAS‐STING pathway (Figure 1). The micronuclear envelope surrounding the cytoplasmic MN is prone to rupture [4], releasing DNA into the cytosol and activating cGAS which produces cyclic GMP‐AMP (cGAMP) [4]. cGAMP activates the stimulator of interferon genes (STING), leading to NF‐κB and IRF3 activation [4]. NF‐κB, once activated, drives transcriptional activation of further pro‐inflammatory cytokines and chemokines [5] and phosphorylated IRF3 induces transcription of Type I interferons [6]. Notably, this relationship could be bidirectional. Genome instability leading to MN formation triggers cGAS‐STING activation and induces an innate inflammatory response [4]. This downstream inflammation could create a “vicious circle,” in which MN formation and cGAS‐STING activation mutually reinforce one another. In the context of cancer, this pathway can stimulate immune responses against cancer cells [7], but may also promote chronic local inflammation, exacerbating tissue DNA damage and early cancer progression [7]. This dynamic interplay is context‐dependent and not currently well understood.

Lymphocyte MN serve as a biomarker for various diseases including chronic kidney disease [8], neurological conditions such as Alzheimer's disease [9], heart failure [10] and some cancers. Elevated MN frequencies (MN%) have been observed in the lymphocytes of patients with haematological cancer [11], lung cancer [12] and colorectal cancer [13]. Furthermore, the International Human Micronucleus (HUMN) project, initiated in 1997, analysed MN data from 20 laboratories and found that individuals with higher baseline lymphocyte MN% faced increased risk of future solid‐cancer development and reduced cancer‐free survival [14].

OAC has emerged as a significant health concern worldwide. Its incidence has steadily increased over recent decades [15] due to the rising prevalence of gastro‐oesophageal reflux disease (GORD), a condition characterised by the reverse flow of stomach contents into the oesophagus. Persistent GORD can progress to the pre‐malignant condition known as Barrett's Oesophagus (BO), where the squamous epithelium of the lower oesophagus is replaced by columnar epithelium [16]. The risk of an individual with BO developing OAC is small yet substantially higher compared to the general population [17]. We hypothesised that the inflammatory environment in early oesophageal carcinogenesis increases MN formation in circulating lymphocytes. This study measured MN in lymphocytes from healthy volunteers and patients with GORD, BO and OAC for the first time. We focused on the biological mechanisms behind observed MN formation, assessing DNA damage susceptibility and repair through ex vivo lymphocyte challenge assays and examined plasma markers of cGAS‐STING activation and oxidative stress. Integrating these analyses, we aimed to clarify the relationship between systemic factors, DNA damage and MN formation in oesophageal cancer development.

## 2 Materials and Methods

### 2.1 Sample Collection

Blood samples were acquired from patients with GORD, BO and OAC attending the local endoscopy department. Patients with GORD were defined as those who had evidence of reflux oesophagitis or those who scored 8 or more in the gastro‐oesophageal reflux disease questionnaire [18]. Patients with BO and OAC had histologically confirmed diseases and had not undergone any chemotherapy. Blood was also collected from consenting healthy volunteers attending the Clinical Research Facility at Swansea University.

**FIGURE 2:**
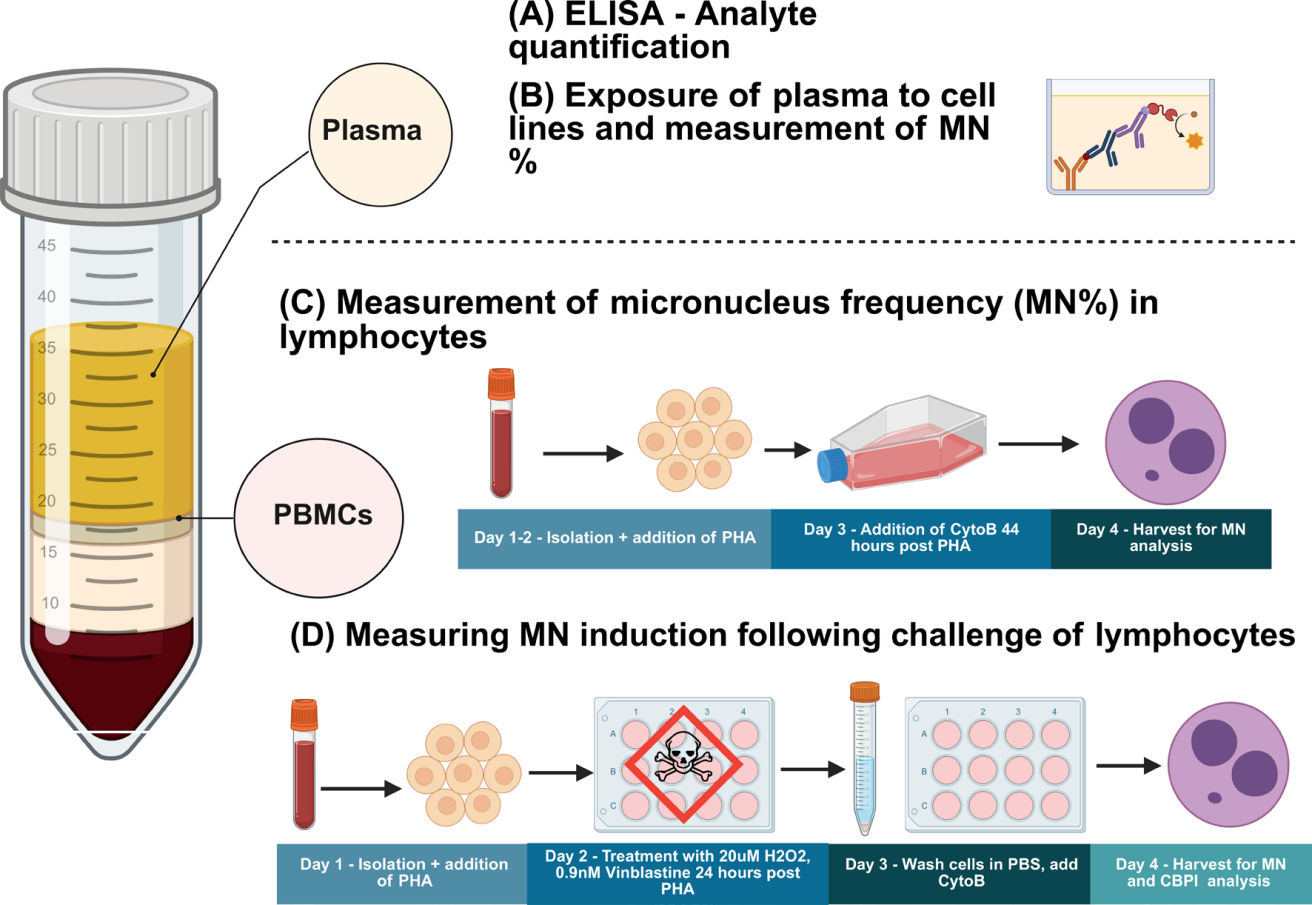
Schematic representation of experiments carried out from a single blood sample. (A) The plasma was used for analyte quantification using enzyme‐linked immunosorbent assays (ELISAs). (B) Patient plasma was used to supplement culture media and treat selected cell lines to assess its ability to induce micronuclei. (C) Peripheral blood mononuclear cells (PBMCs) were isolated from the buffy coat and lymphocyte micronucleus frequency (MN%) measured using the cytokinesis‐block method. (D) Patient lymphocytes were challenged with hydrogen peroxide (H_2_O_2_), DCA and Vinblastine and MN% and cytokinesis block proliferation index (CBPI) measured. Created using BioRender.com.

Blood was collected into lithium heparin tubes (Greiner‐Bio One, Kremsmunster, Austria) and processed within 48 h of sample collection. Biopsies obtained during endoscopy (from patients with BO and OAC only) were immediately stored in RNAlater RNA stabilising reagent (Qiagen, Hilden, Germany) and kept on ice. A schematic representation of experiments carried out from a single blood sample is shown in Figure 2.

### 2.2 Micronucleus Assay

The method used was based on a paper published by Fenech [19]. In short, blood was layered onto an equal volume of Histopaque‐1077 (Sigma‐Aldrich, Missouri, USA) and centrifuged at 400 g for 30 min. The monocyte layer was aspirated and washed twice in phosphate buffer saline (PBS) (Sigma‐Aldrich). The cell pellet was gently resuspended in 1 mL cell culture media (RPMI 1640 with 10% Foetal bovine serum [FBS], 1% l‐glutamine and 1% sodium pyruvate, all Sigma‐Aldrich). Cells were seeded at a density of 1 × 10^6^ cells mL^−1^ in 10 mL of culture media. Phytohaemagglutinin (PHA) (Sigma‐Aldrich) was added at a concentration of 30 mg mL^−1^ to stimulate cell division. After 44 h, Cytochalasin B (CytoB) (Sigma‐Aldrich) was added at a concentration of 4.5 mg mL^−1^ to block cytokinesis. After a further 24–28 h, the lymphocytes were harvested. To assess the impact of PHA treatment, MN were counted in mononucleate cells from the untreated sample and in binucleate cells from the PHA‐treated sample.

### 2.3 Semi‐Automated Scoring With the Metafer System

All centrifuge steps described in this section were as follows: 123 *× g*, 10 min, at 4°C. Cells were first washed in PBS and then pelleted and washed in pre‐warmed 0.56% potassium chloride (KCL) solution. Cells were then resuspended in FIX 1 solution (five‐part methanol, six‐part 0.9% sodium chloride and one‐part acetic acid) for a 10‐min incubation before cells were centrifuged and resuspended in FIX 2 (20% acetic acid in methanol). Cells were incubated at 4°C for 10 min prior to centrifugation. Cells were washed twice in FIX 2, before being stored in FIX 2 at 4°C until slide preparation. Lymphocytes were centrifuged and resuspended in fresh fixative. A 100 μL cell suspension was pipetted onto each slide and left to air dry, then stained with 30 μL VECTASHIELD Antifade Mounting Medium with DAPI (Vector Laboratories, UK) before coverslips were applied. Binucleate cells scored using Metafer (MetaSystems, Altlussheim, Germany). One thousand cells were scored in triplicate per sample. The micronucleus frequency (MN%) was defined as the number of binucleated cells containing one or more micronucleus divided by the total number of binucleated cells (×100).

### 2.4 Centromere Staining

Centromere staining was carried out using StarFish Pan‐Centromeric Chromosome Paint (Cambio, Cambridge, UK) according to the manufacturer's instructions. The FITC‐conjugated pan‐centromeric probe was applied to slides and hybridised for approximately 16 h at 37°C in a humidified chamber. Slides were washed in formamide and saline‐sodium citrate solution and allowed to air dry. DAPI counterstaining was performed using VECTASHIELD Antifade Mounting Medium with DAPI. Centromere status of micronucleated cells was assessed using the Metafer automated scoring system (Zeiss, Jena, Germany). Ten micronucleated cells were scored in triplicate, per individual.

### 2.5 Plasma Analyte Quantification

All plasma samples previously stored at −80°C were thawed to room temperature prior to use. Plasma samples were deproteinised using 5% sulfosalicylic acid prior to analysis. To assess cGAS‐STING pathway activation, plasma levels of 2′3′‐cGAMP (a second messenger produced upon cGAS activation) were quantified using a specific ELISA kit (Cayman Chemicals, Michigan, USA). In addition, 8‐OHdG, a marker of oxidative DNA damage, was measured using the 8‐hydroxy‐2′‐deoxyguanosine ELISA kit (Abcam, Cambridge, UK). Both approaches were carried out according to the manufacturers' guidance.

### 2.6 Challenge Assay

Isolated peripheral blood mononuclear cells (PBMCs) were seeded at a concentration of 1 × 10^6^ cells mL^−1^ and stimulated with PHA (10 μL mL^−1^; 1% v/v) for 24 h to initiate cell proliferation. Cells were then exposed to 20 μM H₂O₂, 0.9 nM vinblastine, or 150 μM deoxycholic acid (DCA) for 20 h. The use of both H_2_O_2_ and DCA in the challenge assay was intended to model two distinct oxidative stress pathways: H_2_O_2_ as a general pro‐oxidant and DCA as a bile acid relevant to oesophageal pathology. The 24‐h pre‐stimulation period was selected to ensure that CytoB could be added at 44 h post‐stimulation, in line with the cytokinesis‐block micronucleus (CBMN) assay protocol described by Fenech [19]. This timing prevented overlap between chemical exposure and CytoB treatment, which can increase cytotoxicity, while maintaining sufficient lymphocyte activation for MN formation. Following chemical treatment, the cells were transferred to fresh media containing CytoB for a further 24 h before harvesting for CBPI and MN scoring as described above.

### 2.7 Tissue Protein Expression

Oesophageal biopsies were placed in 250 mL RIPA buffer and 2.5 mL protease inhibitor cocktail (both Sigma‐Aldrich) and homogenised using 1.4 mm ceramic beads (Cambio). Extracted protein was quantified using the Bio‐Rad DC Protein Assay (Bio‐Rad, Hertfordshire, UK) and Western blotting for inhibitor of NF‐κB (IκB) and β‐actin carried out. Antibodies against IκB and β‐actin (Cell Signalling Technology, Massachusetts, USA) were diluted 1:1000 and incubated at 4°C overnight. Membranes were washed and incubated with horse‐radish peroxidase conjugated secondary antibodies (diluted 1:1000) (Cell Signalling Technology) at room temperature for 1 h. The immune‐star Western C chemiluminescence kit (Bio‐Rad) was used to detect proteins of interest and protein bands detected using a ChemiDoc XRS (Bio‐Rad).

### 2.8 Statistical Analysis

Statistical analysis was carried out using IBM SPSS Statistics v29 and GraphPad Prism v10. MN frequency data were log‐transformed to address skewness. For comparison of histology groups, a General Linear Model was used to explore the relationship between demographic and clinical factors and MN frequency in 155 patients. Age, gender, body mass index (BMI), smoking status and diagnosis category were included as independent variables and covariates to adjust for potential confounding in MN% comparisons between groups and sexes. Planned contrasts were used to explore differences between histology categories in the presence of confounders. Statistical significance, effect sizes and confidence intervals were reported for all variables in the final model. Due to non‐normality in key variables, non‐parametric tests were employed throughout. For continuous variables, the Kruskal–Wallis test was used to test for differences between groups.

## 3 Results

### 3.1 Lymphocyte MN% Was Linked to Age, BMI and Histology in Study Participants

To explore the mechanistic link between MN formation and histology, 170 participants were recruited to the study (32 healthy volunteers, 51 patients with GORD, 46 patients diagnosed with BO and 41 OAC patients). The characteristics of each group are included in Table S1. To explore the origin of lymphocyte MN and the potential role of PHA stimulation in “fixing” existing DNA lesions, MN% was measured both before and after PHA stimulation. Figure S1 shows PHA's mitogenic effect was required for unrepaired DNA lesions to manifest as MN ex vivo.

**FIGURE 3:**
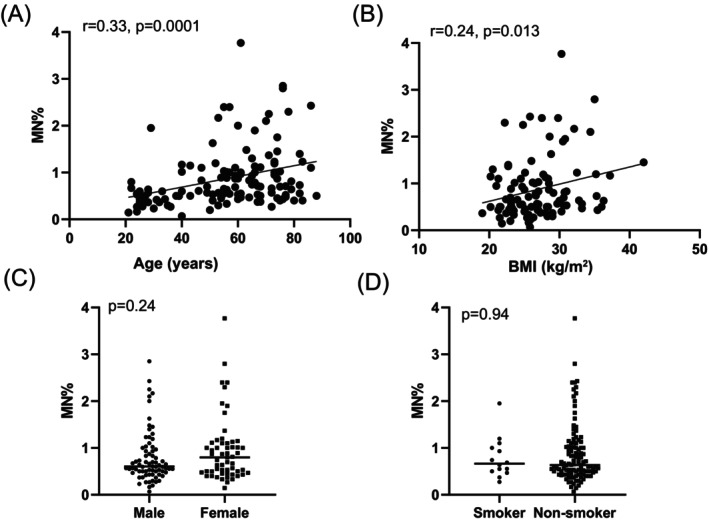
Demographic influences on lymphocyte micronucleus frequency (MN%) in non‐cancer participants. (A) A positive correlation between increasing age and MN% (*r* = 0.33, *p* < 0.001) and (B) Increasing body mass index (BMI) and MN% were observed (*r* = 0.24, *p* = 0.013). (C) No difference in MN% between males and females (*p* = 0.24). (D). No difference in MN% between smokers and non‐smokers (*p* = 0.94).

The non‐cancer cohort (*n* = 129) was analysed to assess demographic effects on lymphocyte MN% (Figure 3). Linear regression showed MN% increased with age (*p* < 0.0001) and BMI (*p* = 0.013) (Figure 3A,B). No significant difference in MN% was found between males and females (*p* = 0.24) or smokers and non‐smokers (*p* = 0.94), although only 14 participants self‐reported as smokers (Figure 3C,D).

**FIGURE 4:**
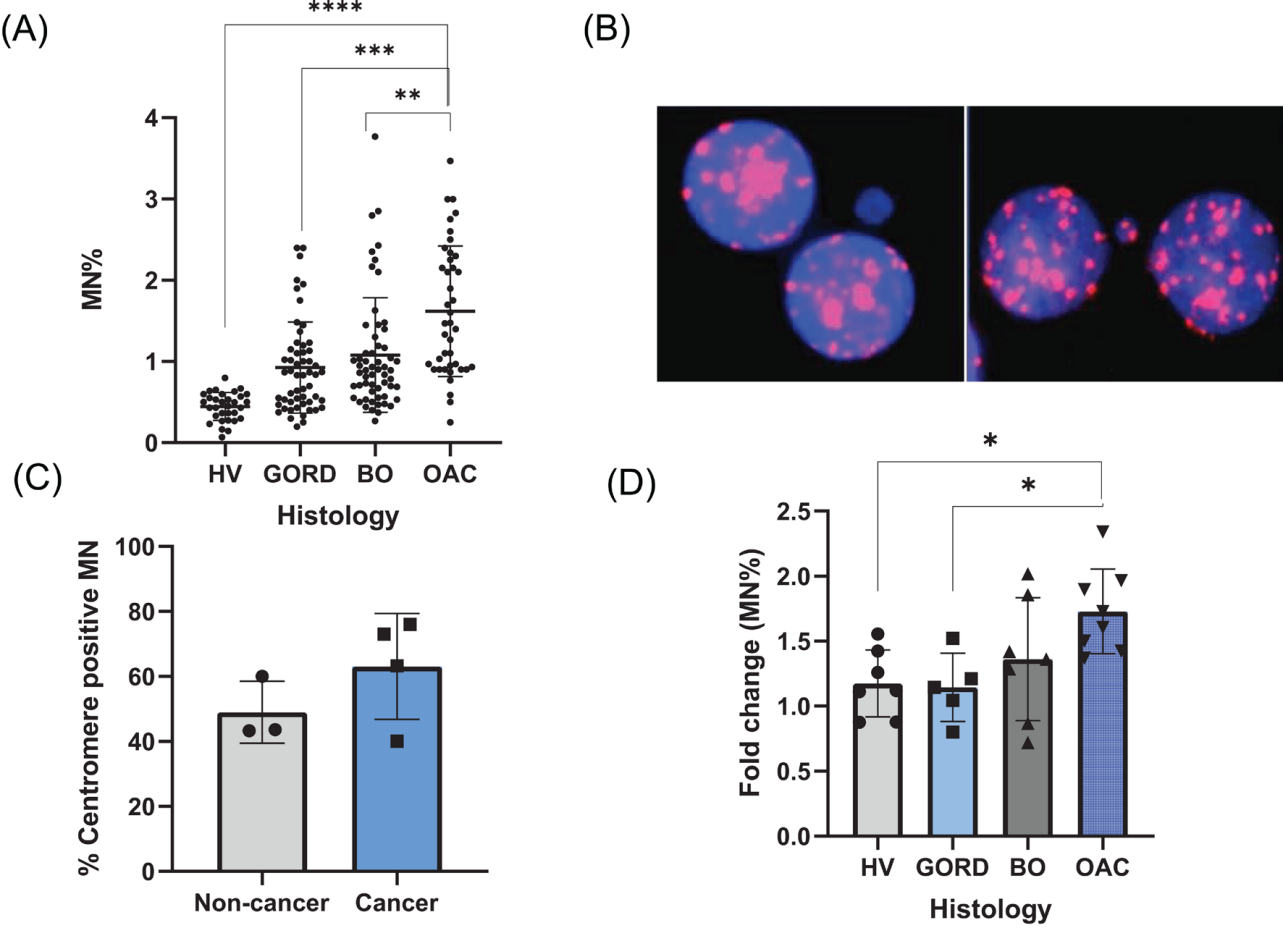
Aneugenic mechanisms of lymphocyte MN formation. (A) Lymphocyte micronucleus frequency (MN%) for healthy volunteers (HV) (*n* = 32) and patients with gastro‐oesophageal reflux disease (GORD) (*n* = 51), Barrett's oesophagus (BO) (*n* = 46) and oesophageal adenocarcinoma (OAC) (*n* = 41). OAC patients exhibited higher MN% compared to HVs (*p* < 0.001), GORD patients (*p* < 0.001) and BO patients (*p* < 0.001). (B) Representative fluorescent microscopy images of a centromere positive MN (left panel) and centromere negative MN (right panel). (C) Quantification of the percentage of the centromere status of MN for non‐cancer (*n* = 3) and cancer patients (*n* = 4) (*p* = 0.24). (D) OAC patient lymphocytes had higher MN induction post treatment with 0.9 nM vinblastine compared to HV's (*p* = 0.025) and patients with GORD (*p* = 0.033). Error bars represent standard deviation.

This study investigated the baseline frequency of MN across histological groups, examined the centromere status of MN in a small subset of samples and assessed susceptibility to aneugen‐induced damage (Figure 4). When comparing between histology groups OAC patients had elevated MN%, with a median lymphocyte MN% of 1.43% (95% CI: 1.34%–1.86%), significantly higher than BO patients (0.87%, 95% CI: 0.86%–1.38%, *p* < 0.001), GORD patients (0.87%, 95% CI: 0.78%–1.12%, *p* < 0.001) and healthy volunteers (0.47%, 95% CI: 0.38%–0.51%, *p* < 0.001) (Figure 4A). A multivariate generalised linear model confirmed a highly significant association between histology and MN%.

### 3.2 OAC Lymphocytes Show Altered Sensitivity to Aneugenic Challenge

To explore whether aneugenic mechanisms may contribute to elevated MN% in OAC patient lymphocytes, MN centromere status was assessed using FISH probes (Figure 4B). MN positive for centromeres were considered to contain whole chromosomes. Cancer patients showed a trend toward an increased number of centromere‐positive MN compared to non‐cancer patients, but this difference was not significant (*p* = 0.24, Figure 4C). Lymphocytes from all of the histological groups were then isolated and treated with the aneugenic agent vinblastine in vitro as part of a challenge assay. OAC patient lymphocytes showed increased susceptibility to vinblastine‐induced MN formation compared to healthy controls (*p* = 0.025) and GORD patients (*p* = 0.033), with Barrett's patients showing intermediate susceptibility (Figure 4D).

### 3.3 Association Between Oxidative DNA Damage and MN Formation in Lymphocytes

**FIGURE 5:**
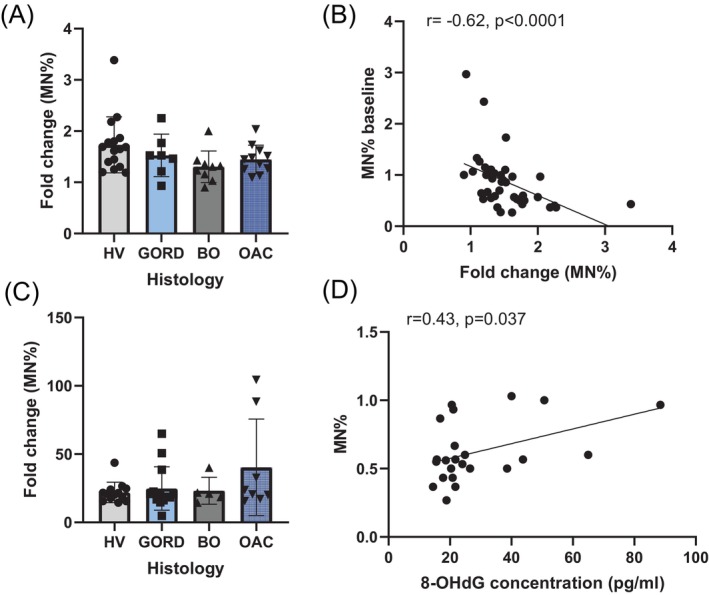
Oxidative stress mechanisms of lymphocyte micronucleus (MN%) formation. (A) Fold change in MN induction following treatment of lymphocytes with 20 μM H_2_O_2_ from healthy volunteers (HV) and patients with gastro‐oesophageal reflux disease (GORD), Barrett's oesophagus (BO) and oesophageal adenocarcinoma (OAC). (B) Comparison of MN fold change following treatment of lymphocytes with 20 μM H_2_O_2_ with baseline MN levels. Individuals with higher baseline MN% were less sensitive to H_2_O_2_ induced MN formation (*n* = 41, *r* = −0.62, *p* < 0.001). (C) OAC patients appeared to have elevated levels of 8‐hydroxy 2‐deoxyguanosine (8‐OHdG) in their plasma, but this difference was not statistically significant (*p* = 0.75). (D) Individuals with higher MN% had higher levels of plasma 8‐OHdG (*n* = 23, *r* = 0.43, *p* = 0.037). Error bars represent standard deviation.

Next, this study aimed to explore the link between MN% and oxidative stress (Figure 5). To determine if histology‐linked elevation in MN% was due to oxidative stress and DNA damage, lymphocytes were challenged with the pro‐oxidant clastogen H_2_O_2_. No obvious differences in susceptibility to H_2_O_2_ were seen across all histological groups (Figure 5A). However, comparing MN fold change after H_2_O_2_ treatment to initial MN% in untreated lymphocytes for 41 participants revealed a strong negative correlation (*R* = −0.62, *p* < 0.0001) (Figure 5B). Similar results were observed with the pro‐oxidant bile acid DCA; no histology‐dependent differences in MN induction were detected, but a negative correlation existed between untreated MN% and MN% fold change after DCA treatment (*R* = −0.50, *p* = 0.007, *n* = 27) (Figure 2). This negative correlation was absent in vinblastine‐challenged lymphocytes. Given these interesting findings, plasma levels of 8‐hydroxy‐2′‐deoxyguanosine (8‐OHdG), a marker of oxidative DNA damage, were measured in 23 individuals. OAC patients showed elevated 8‐OHdG compared to non‐cancer individuals, though this was not statistically significant versus BO, GORD, or healthy volunteers (*p* = 0.757) (Figure 5C). A positive correlation was found between lymphocyte MN% and plasma 8‐OHdG concentration (*R* = 0.43, *p* = 0.037) (Figure 5D), suggesting an association between circulating oxidative stress and MN formation.

### 3.4 Activation of the cGAS‐STING Pathway Was Linked to MN Formation

**FIGURE 6:**
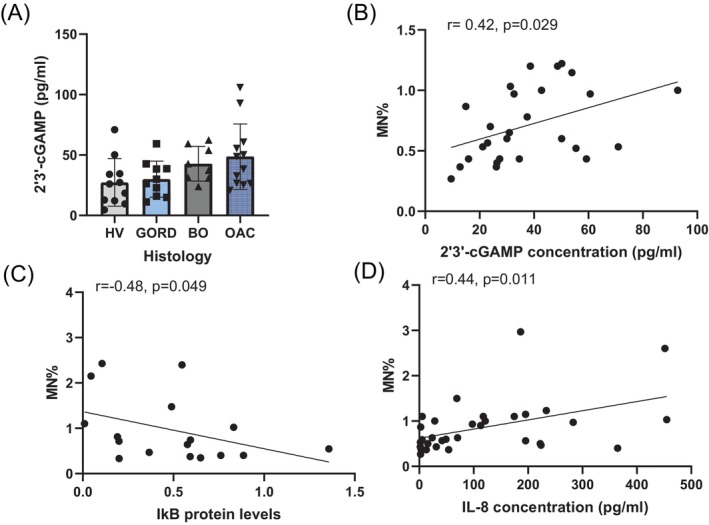
Investigation of the relationship between lymphocyte MN and the cGAS‐STING pathway. (A) Quantification of 2′3′‐cyclic GMP‐AMP (cGAMP) in the plasma of healthy controls and patients with different oesophageal histologies. (B) There was a significant positive correlation between increasing lymphocyte MN% and higher cGAMP in patient plasma (*n* = 27, *r* = 0.42, *p* = 0.029). (C) Individuals with higher lymphocyte MN% had lower levels of IKB protein in the oesophageal tissue (hence higher levels of NF‐kB activity) (*R* = −0.48, *p* = 0.049). (D) There was a significant positive correlation between plasma IL‐8 levels and lymphocyte MN% (*R* = 0.196, *p* = 0.0111). Error bars represent standard deviation.

Due to the suggested link between oxidative stress and lymphocyte MN%, we further explored inflammation's role via the cGAS‐STING and NF‐kB pathways (Figure 6). Using ELISA, we measured plasma 2′3′‐cGAMP levels in all histological groups (Figure 6A,B). Although levels tended to be higher in OAC patients, this was not significant (*p* = 0.057). However, lymphocyte MN% positively correlated with individual plasma 2′3′‐cGAMP concentrations (*R* = 0.42, *p* = 0.029) (Figure 6B). As cGAS‐STING activates NF‐kB, we measured oesophageal tissue IkB protein by Western blot in all groups except for the healthy volunteers. Higher lymphocyte MN% corresponded to significantly lower IkB protein levels, indicating increased NF‐kB activity (*p* = 0.049) (Figure 6C). Additionally, across all groups, lymphocyte MN% positively correlated with plasma IL‐8, a downstream NF‐kB chemokine (*p* = 0.0111) (Figure 6D). These findings suggest lymphocyte MN formation is linked to inflammation in tissue and blood.

## 4 Discussion

Here for the first time, we assessed the mechanistic basis for lymphocyte MN% levels in patients diagnosed with oesophageal disease (GORD, BO and OAC). We have shown, using a comprehensive study design, that MN% is elevated in OAC patients (independent of confounders like age) and that MN formation is linked to oxidative stress, inflammation and the cGAS‐STING pathway with a mechanism influenced by the PHA stimulation of lymphocytes fixing DNA lesions as double strand breaks.

In the non‐cancer group, sex‐based differences in MN% were absent, results consistent with a larger population‐based study by Murgia and colleagues. Age showed a positive correlation with MN%, aligning with previous non‐cancer cohort studies [20] and mirroring erythrocyte PIG‐A mutation data in a similar cohort of oesophageal disease patients [21]. This age‐related effect is influenced by acquired somatic mutations, extended mutagen exposure and age‐related defects in DNA repair and the mitotic machinery leading to chromosome mis‐segregation [22]. No difference in MN% was observed between smokers and non‐smokers, potentially influenced by the small number of smokers in this cohort (*n* = 14). Whilst some studies suggest the lymphocyte MN assay is sensitive to cigarette‐smoke polycyclic aromatic hydrocarbons (PAH's), the HUMN project involving 5710 subjects found no difference in MN% between smokers or non‐ or former‐smokers [23]. We found a positive correlation between increasing MN% and BMI in the non‐cancer group. This supports previous reports of an increase in MN% in those with metabolic syndrome [24].

This is the first study that has attempted to understand the mechanisms behind increased MN% formation in the lymphocytes of cancer patients (here with OAC as a model). Interestingly, we have clearly shown that OAC patients have elevated MN% in circulating lymphocytes compared to healthy volunteers, GORD and BO patients even when accounting for confounding factors such as age. OAC is an inflammatory driven cancer, and evidence suggests MN formation may play an active role in promoting inflammation through pathways such as cGAS‐STING [25]. Previous studies measuring MN% in the oesophageal mucosa of young people (age 15–26 years) found no association between increased MN% and degree of oesophagitis [26]. Moreover, Karaman and colleagues found that patients with Barrett's oesophagus had elevated lymphocyte MN% compared to matched controls [27], although the levels of MN% were much higher than those reported here. The elevation of MN% in OAC lymphocytes as shown here offers the possibility of its use in monitoring of disease and the mechanistic basis for MN formation offers novel therapeutic opportunities.

To explore whether elevated MN levels in OAC patient lymphocytes involved aneugenic events, we assessed MN centromere status in a small subset of samples. Although this result was not statistically significant, there was a trend toward more MN containing whole chromosomes in OAC patients. Lymphocytes from OAC patients were also more sensitive to the aneugenic agent vinblastine, indicating increased susceptibility to defects in chromosome segregation. These findings support the idea that lymphocytes can reflect systemic genomic instability, including that observed in the primary tumour. This is consistent with reports of aneuploidy in oesophageal epithelial cells and in lymphocytes of patients with other cancers [28, 29].

With the knowledge that OAC is an inflammatory‐driven cancer, we investigated the role of oxidative stress as a potential mechanism behind elevated lymphocyte MN levels. Following exposure to the pro‐oxidants H_2_O_2_ and DCA, individuals with higher baseline MN% exhibited a smaller fold increase in MN formation. Although this observation does not demonstrate a causal adaptive mechanism, several explanations are plausible. Prior studies have shown that repeated genotoxic stress can upregulate DNA repair genes and antioxidant responses, reducing the magnitude of subsequent DNA damage [30]. This has been demonstrated following chronic low‐level H_2_O_2_ exposure, whereby cells increased oxidative DNA repair capacity [31] and after repeated exposure to the carcinogen benzo(a)pyrene diol epoxide (BPDE), which induced sustained upregulation of nucleotide excision repair genes [32]. Kumar and colleagues reported that men occupationally exposed to ionising radiation had higher baseline lymphocyte MN% [33], as well as elevated plasma antioxidant markers, further supporting the idea that pre‐existing DNA damage can influence susceptibility to further oxidative stress. The reduced MN fold‐change in individuals with higher baseline MN% may therefore reflect pre‐existing cellular adaptations or differences in DNA repair capacity rather than reflecting a true protective effect, as these cells still have higher absolute MN% but show a smaller inducible response in vitro. Chronic inflammation is known to induce DNA damage through the production of reactive oxygen and nitrogen species (RONS), generated predominantly by activated immune cells such as neutrophils and macrophages [34]. These cells produce superoxide and nitric oxide, which undergo further reactions to form additional RONS, including hydroxyl radicals, hydrogen peroxide and peroxynitrite, capable of damaging DNA in surrounding cells [34]. While nitrative DNA lesions such as 8‐nitroguanine are considered more specific markers of inflammation‐mediated DNA damage [35], oxidative lesions such as 8‐hydroxy‐2′‐deoxyguanosine (8‐OHdG) are widely reported in gastrointestinal diseases characterised by chronic inflammation and mucosal oxidative stress [36]. Importantly, oxidative DNA lesions including 8‐OHdG have been reported in Barrett's oesophagus and oesophageal adenocarcinoma tissues in the context of inflammation‐related carcinogenesis [37].

Quantification of 8‐OHdG in this study showed that, whilst there was no significant difference across the histological series, individuals with higher MN% exhibited significantly increased levels of plasma 8‐OHdG. We hypothesise that lymphocytes migrating through an inflammatory gastrointestinal environment are exposed to RONS, which are known to cause oxidative DNA lesions [38]. These lesions may persist and upon mitotic stimulation with PHA ex vivo become expressed as MN. Previous reports have also suggested that the formation of MN ex vivo is due to persistent damage obtained in vivo that is not repaired before cells are later driven to proliferate [39]. In addition to reflecting oxidative DNA damage, 8‐OHdG may indirectly contribute to MN formation through the activation of caspases and endonucleases [40].

Our recent findings on the ability of human plasma to induce MN in vitro support the concept of circulating DNA damaging agents (potentially RONS), driving genome instability in blood cells [41].

The second messenger 2′3′‐cGAMP forms when double‐stranded DNA, including DNA derived from MN, binds to and activates cGAS. In our study, we observed a correlation between lymphocyte MN% and plasma 2′3′‐cGAMP, which is consistent with the idea that MN formation may contribute to increased cGAMP production. Previous work indicates that 2′3′‐cGAMP can be exported into the extracellular space or taken up by other cells, although it is rapidly degraded by ENPP1 [42]. We also identified a negative correlation between lymphocyte MN% and IkB levels in oesophageal tissue, which suggests a potential relationship between systemic MN induction and local NF‐kB activity. IkB is the inhibitor of NF‐kB and is known to be degraded through several mechanisms, including activation of the cGAS‐STING pathway. NF‐kB translocation to the nucleus and the induction of target genes such as IL‐8 may be particularly relevant in the OAC model. NF‐kB activity and IL‐8 production are known to increase as tissue progresses from squamous epithelium to Barrett's mucosa and then to adenocarcinoma [43] and DCA, a bile constituent, has been shown to increase MN formation in vitro as well as activate NF‐kB and IL‐8 production [44].

It is important to note, however, that the biomarkers examined here were measured in different biological matrices. MN were assessed in circulating lymphocytes, 2′3′‐cGAMP in plasma, and NF‐kB, IkB and IL‐8 in oesophageal tissue or plasma. These findings should therefore be interpreted as parallel indicators of genotoxic and inflammatory processes rather than direct mechanistic links. Plasma 2′3′‐cGAMP may reflect broader cGAS activity in the body, and the relationship between lymphocyte MN% and tissue IkB levels may indicate that systemic genotoxic stress corresponds with local inflammatory signalling. As these measurements arise from different cell types, further studies using matched samples from the same tissue sources will be necessary to clarify the nature of these relationships.

## 5 Conclusion

This study provides new insight into the biology of circulating blood‐cell genomic instability across the GORD, BO and OAC spectrum. We show that lymphocyte MN% is elevated in individuals with OAC compared with non‐cancer controls, independent of confounders such as age. Several processes, including oxidative stress, oxidative DNA damage and aneugenic mechanisms, appear to be associated with increased MN formation.

Individuals with higher baseline MN% showed reduced MN induction after oxidative challenge, which may reflect underlying differences in cellular stress responses or DNA repair capacity. Plasma 8‐OHdG levels were associated with MN%, suggesting a link between systemic oxidative DNA damage and chromosomal instability. Associations were also observed between MN%, plasma 2′3′‐cGAMP and oesophageal tissue IkB levels, indicating connections between systemic genomic instability and activation of pathways such as cGAS‐STING and NF‐kB. As these measurements were made in different biological matrices, they cannot be interpreted as causal relationships.

Overall, these findings support the idea that lymphocyte MN reflects broader inflammatory and genomic‐instability processes relevant to OAC. Further work is now underway to clarify the relationship between systemic MN formation and malignant transformation in the oesophagus, with the aim of identifying potential biomarkers and therapeutic targets.

## Author Contributions

**Kathryn Munn:** methodology, writing – review and editing, writing – original draft, formal analysis, data curation, investigation. **Rachel Lawrence:** writing – original draft, methodology, formal analysis, data curation, investigation, writing – review and editing. **Hasan Haboubi:** methodology, investigation. **Hamsa Naser:** investigation. **Kate Hurlow:** investigation. **Ume‐Kulsoom Shah:** methodology. **Lisa Williams:** investigation. **Sarah Gwynne:** investigation. **Owen Bodger:** formal analysis. **Jiri Zavadil:** writing – review and editing. **Francois Virard:** writing – review and editing. **Shareen Doak:** supervision. **Laura E. Thomas:** writing – review and editing. **Gareth Jenkins:** conceptualization, supervision, funding acquisition, writing – review and editing.

## Funding

This study was financially supported by Cancer Research Wales (through a PhD studentship to RL and KH, plus multiple project grants) and Swansea University (through a PhD studentship to K.M.). Study sponsorship was obtained from Swansea Bay University Health Board Research and Development department.

## Ethics Statement

Ethical approval for this study was obtained from the South‐West Wales Local Research Ethics Committee (REC reference: 11/WA/0367) and South‐West Wales Research Ethics Committee 6 (REC reference: 13/WA/0190). Healthy volunteers were recruited under the research study given a favourable ethical opinion by Swansea University Medical School (SUMS) Research Ethics committee (REC), project reference 2022‐0029. All participants provided written informed consent prior to inclusion in the study. The research was conducted in accordance with the Declaration of Helsinki.

## Conflicts of Interest

The authors declare no conflicts of interest.

## Supporting information


**Table S1:** Demographic and clinical characteristics of study participants across disease groups (median values and 95% confidence intervals).
**Figure S1:** Micronucleus frequency (MN%) of matched patient lymphocytes pre‐ and post‐stimulation with phytohemagglutinin (PHA). DNA lesions are more commonly converted to micronuclei (MN) following ex vivo stimulation with PHA.
**Figure S2:** Lymphocyte MN% fold change following treatment with deoxycholic acid (DCA). (A) MN% fold change following treatment for the different histologies. (B) Comparison of MN fold change following treatment with baseline MN levels. Individuals with higher baseline MN% were less sensitive to DCA induced MN formation (*r* = −0.5, *p* = 0.007).

## Data Availability

Data will be made available upon request to the corresponding author.
