# Supplementary material for: Lymphocyte Micronucleus Formation Is Driven by Inflammation‐Induced Oxidative DNA Damage in Oesophageal Cancer Development
Source: Int J Cancer. 2026 Apr 25;159(5):1311–21. doi: 10.1002/ijc.70494 (PMC13340993; doi:10.1002/ijc.70494)
Supplement: Supplementary file 1 — Table S1: Demographic and clinical characteristics of study participants across disease groups (median values and 95% confidence intervals). Figure S1: Micronucleus frequency (MN%) of matched patient lymphocytes pre‐ and post‐stimulation with phytohemagglutinin (PHA). DNA lesions are more commonly converted to micronuclei (MN) following ex vivo stimulation with PHA. Figure S2: Lymphocyte MN% fold change following treatment with deoxycholic acid (DCA). (A) MN% fold change following treatment for the different histologies. (B) Comparison of MN fold change following treatment with baseline MN levels. Individuals with higher baseline MN% were less sensitive to DCA induced MN formation (r = −0.5, p = 0.007). [file IJC-159-1311-s001.pdf]

Title: **Lymphocyte Micronucleus Formation Is Driven by Inflammation-Induced Oxidative DNA Damage in Oesophageal Cancer Development**

Short title: **Lymphocyte micronuclei in oesophageal cancer**

Authors: **Kathryn Munn, Rachel Lawrence, Hasan Haboubi, Hamsa Naser, Kate Hurlow, Ume-Kulsoom Shah, Lisa Williams, Sarah Gwynne, Owen Bodger, Jiri Zavadil, Francois Virard, Shareen Doak, Laura E Thomas, Gareth Jenkins**

Table of Contents

- Supplementary Table 1. Demographic and clinical characteristics of study participants across disease groups (median values and 95% confidence intervals)
- Supplementary Figure 1. Micronucleus frequency (MN%) pre- and post-stimulation with phytohemagglutinin
- Supplementary Figure 2. Lymphocyte MN% fold change following deoxycholic acid treatment

Supplementary Table 1. Demographic and clinical characteristics of study participants across disease groups (median values and 95% confidence intervals)

Characteristics (Median value)	Healthy volunteers (n=32)	GORD (n=51)	BO (n=46)	OAC (n=41)
Average age (y) (95% CI)	28 (25-33)	60 (57-66)	68 (51-82)	72 (52-85)
Gender, % male	50% (16/32)	49% (25/51)	65.2% (30/46)	87.8% (36/41)
BMI (kg/m^2) (95% CI)	24.6 (23.1-27)	26.5 (25.1-29.8)	27 (25.8-32.1)	24.7 (22-26.8)
Smoking, % use	9.3% (3/32)	4.2% (2/51)	19.4% (9/46)	20% (8/41)
MN% (95% CI)	0.47% (0.38-0.51%)	0.87% (0.78-1.12%	0.87% (0.86-1.38%)	1.43% (1.34-1.86%)

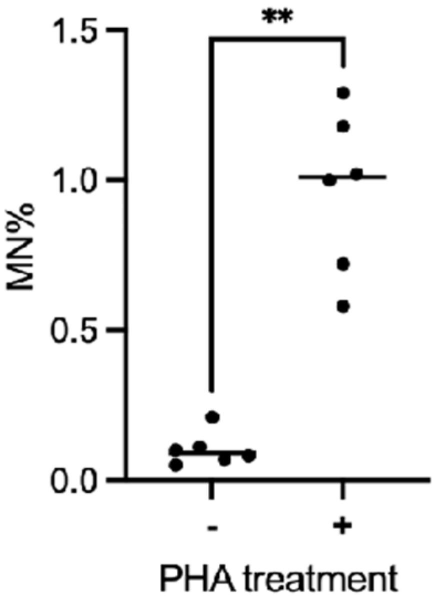

Supplementary Figure 1: Micronucleus frequency (MN%) of matched patient lymphocytes pre- and post- stimulation with phytohemagglutinin (PHA). DNA lesions are more commonly converted to micronuclei (MN) following *ex vivo* stimulation with PHA.

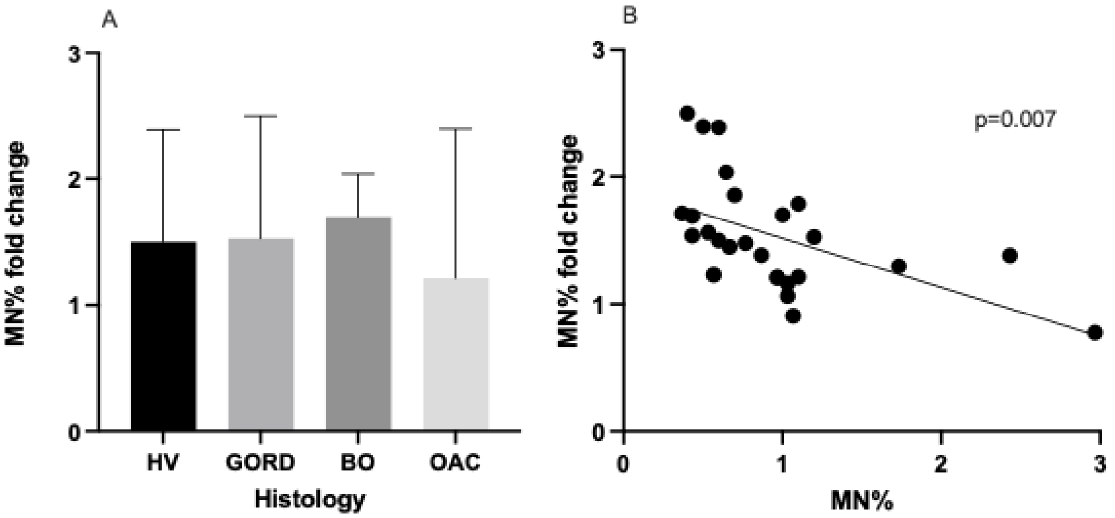

Supplementary Figure 2: Lymphocyte MN% fold change following treatment with deoxycholic acid (DCA). (A) MN% fold change following treatment for the different histologies. (B). Comparison of MN fold change following treatment with baseline MN levels. Individuals with higher baseline MN% were less sensitive to DCA induced MN formation (r=-0.5, p=0.007).
